# The December 2015 North Pole Warming Event and the Increasing Occurrence of Such Events

**DOI:** 10.1038/srep39084

**Published:** 2016-12-15

**Authors:** G. W. K. Moore

**Affiliations:** 1 Department of Physics University of Toronto, Toronto, Ontario Canada

## Abstract

In late December 2015, widespread media interest revolved around forecasts that the surface air temperature at the North Pole would rise above freezing. Although there has been significant interest in the enhanced warming that is occurring at high northern latitudes, a process known as arctic amplification, remarkably little is known about these midwinter warming events at the pole including their frequency, duration and magnitude as well as the environmental conditions responsible for their occurrence. Here we use buoy and radiosonde data along with operational weather forecasts and atmospheric reanalyses to show that such events are associated with surface cyclones near the pole as well as a highly perturbed polar vortex. They occur once or twice each decade with the earliest identified event taking place in 1959. In addition, the warmest midwinter temperatures at the North Pole have been increasing at a rate that is twice as large as that for mean midwinter temperatures at the pole. It is argued that this enhanced trend is consistent with the loss of winter sea ice from the Nordic Seas that moves the reservoir of warm air over this region northwards making it easier for weather systems to transport this heat polewards.

Media attention in late December 2015 was focused on the prospect that the surface air temperature at the North Pole would rise above freezing^1^,^2^. This event was reported to be unprecedented in the instrumental record of the International Arctic Buoy Program (IABP)^3^,^4^. It was the final extreme weather event in a year that had many such events and reports of causal connections between the warming event at the pole and severe weather that occurred during the preceding days in Texas and the United Kingdom were made^5^,^6^.

Mean temperatures in the Arctic have been warming at an accelerated rate as compared to the rest of the Northern Hemisphere, a process known as arctic amplification^7^,^8^. Positive feedbacks in the climate system associated with the loss of sea ice are thought to be responsible for this amplification^7^,^9^,^10^. This regional warming has resulted in modifications to the large-scale atmospheric circulation that have led to changes in the planetary wave field^11^. One consequence of these changes is hypothesized to be an enhanced waviness of the polar vortex, the region in the upper atmosphere where cold polar air is situated and whose boundary is the jet stream. This change in the upper-atmospheric circulation has been linked to more extreme mid-latitude weather including cold air outbreaks that are associated with the southward extension of the polar vortex^7^. There is however considerable debate concerning this linkage^12^,^13^.

Although the focus of current research on the connection between extreme weather and perturbations of the polar vortex has been on the impacts in mid-latitudes^7^,^9^,^12^, there are also instances in which the development of the planetary wave field results in the jet stream being displaced northwards leading to a ‘pinching’ of the polar vortex and a warming at high northern latitudes^14^. These events have been shown to be associated with the advection of moisture into the Arctic that is most pronounced in the Atlantic sector where is it typically associated with the presence of a low-pressure system over the Greenland Sea^15^,^16^. Longwave radiative effects associated with the increased concentration of water vapour and cloud formation have been shown to enhance the surface heating during these events^15^,^17^.

As we shall show, the late December 2015 warming event at the pole was associated with a perturbed polar vortex that advected warm and moist air into the North Pole region from the adjoining Nordic Seas. Indeed, forecasts suggested that it was raining at the pole during this event. These so-called ‘rain on snow’ events can cause significant stresses on Arctic sea ice, infrastructure and ecosystems^18^,^19^,^20^,^21^. We will also show that these midwinter warming events occur once or twice each decade with an event in 2014 as well as one in 1959. The identification of events prior to 1959 is hampered by the lack of a comprehensive upper-air observing network prior to this time^22^. In addition, the warmest midwinter surface air temperatures at the pole are increasing at a rate that is twice as high as that for the mean midwinter surface air temperatures indicating that these events may become more common in the future.

## Results

**Figure 1:**
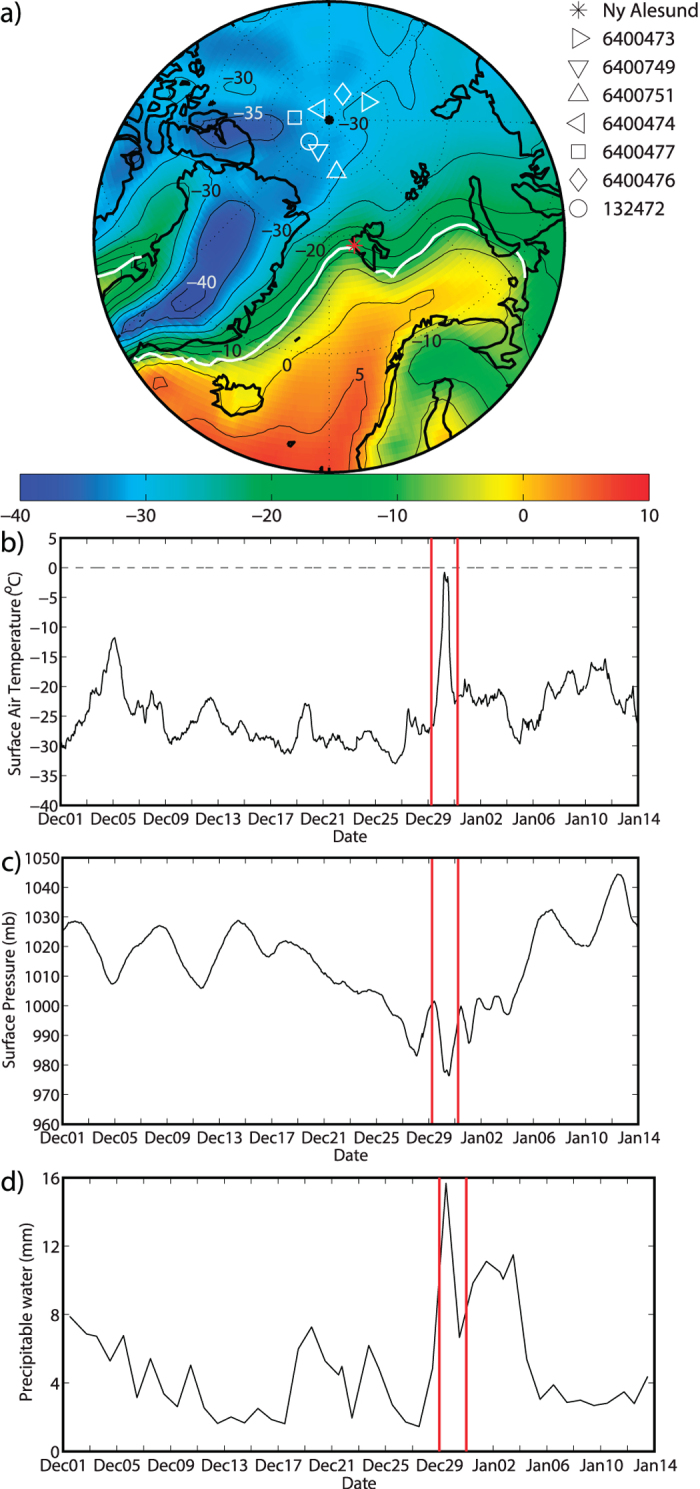
Climatological mean and midwinter 2015/2016 meteorological conditions in the vicinity of the North Pole. (**a**) The December mean surface air temperature (^o^C- shading and contours) from the NCEP Reanalysis 1958–2015 with the 50% sea ice concentration indicated by the thick white contour. The locations of the 7 meteorological buoys situated within 500 km of the pole during this period are indicated as is the location of the radiosonde station at Ny Alesund. The: (**b**) surface air temperature (^o^C) and (**c**) surface pressure (mb) at the North Pole during midwinter 2015/2016 based on the data from these meteorological buoys. (**d**) The precipitable water (mm) based on radiosonde data from Ny Alesund. The period of the late December 2015 warming is indicated by the red lines in (**b–d**). Figure produced using MATLAB R2013b (http://www.mathworks.com).

Although the North Pole has a mean surface air temperature close to −30 °C during the month of December, it is nevertheless adjacent to a region with a large meridional temperature gradient associated the transition from sea ice to open water that occurs in the vicinity of Fram Strait and Barents Sea (Fig. 1). Indeed on average during December, the 0 °C isotherm is situated over the Greenland and Barents Seas within 2000 km of the pole. Figure 1 also shows time series of the surface air temperature and pressure at the North Pole during December 2015 and early January 2016 as determined from the 7 IABP meteorological buoys that were in the vicinity of the pole during this period. The time series of precipitable water, the vertical integral of the specific humidity and a useful field for diagnosing the long-range transport of atmospheric moisture^23^, at Ny Alesund, the closest radiosonde site to the pole, is also shown. Please refer to the Methods for additional details.

For most of December 2015, the North Pole had a surface air temperature of ~−25 °C with a number of small amplitude warming events that were associated with passing cyclones. Over the 24 hour period from 06 UTC 29 December to 06 UTC 30 December, the surface air temperature at the North Pole rose from −26.8 °C to −0.8 °C. Based on the characteristics of the IABP buoys, the uncertainty in the surface air temperature at the pole is on the order of +/− 0.5 °C^24^. This warming event was associated with a drop in surface pressure of ~24 mb. One day later, the surface air temperature had returned to more seasonal values that persisted through mid January 2016. The time series of precipitable water at Ny Alesund indicates that there were a number of events where there was a local maxima in this field with the largest occurring at 12 UTC on 29 December, approximately 18 hours before the maximum surface air temperature observed at the pole. Supplementary Figure 1 shows the vertical profiles of temperature and humidity from this sounding indicating that the thermal and moisture anomalies were largest at the surface with secondary maxima at ~2 km. The values attained during this event exceeded the 99^th^ percentile values for the midwinter period throughout the lower troposphere.

**Figure 2:**
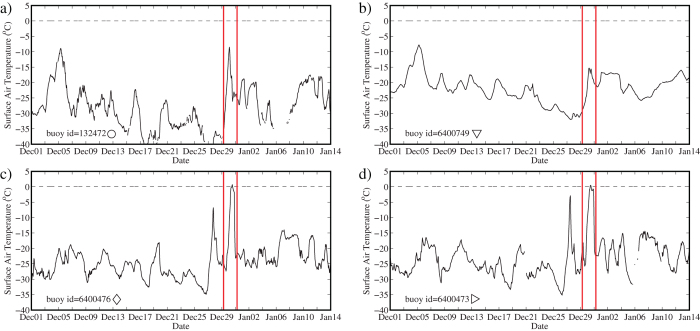
Time series of: surface air temperature (^o^C) at selected meteorological buoys in the vicinity of the North Pole during the 2015 midwinter. The period of the 2015 warming is indicated.

There was considerable variability amongst the various buoys with respect to the magnitude of the warming observed during this event. Examples of this variability are presented in Fig. 2. The buoys with ids 132472 and 6400749 were only 120 km apart on 30 December (Fig. 1). Even though they were in close proximity, the warming at buoy 132472 was more pronounced than that for the buoy 6400749. The buoys with ids 6400473 and 6400476 were farthest from Fram Strait and yet experienced surface air temperatures of 0.5+/−0.5 °C and 0.7+/−0.5 °C respectively on the 30^th^ as well as a distinct more modest warming on the 28^th^. The southernmost buoy with id 6400751 attained a surface air temperature of 2.2+/− 0.5 °C during the event. A common characteristic amongst all the buoys was the short duration of the warming event.

To investigate the spatial variability identified in Fig. 2, meteorological fields from the final operational analysis from the National Centers for Environmental Prediction (NCEP) Global Data Assimilation System^25^ (FNL-GDAS) were examined as well as those from the JRA55^26^ and NCEP^27^ and ERA-I^28^ Reanalyses. Please refer to the Methods for additional information on these datasets.

**Figure 3:**
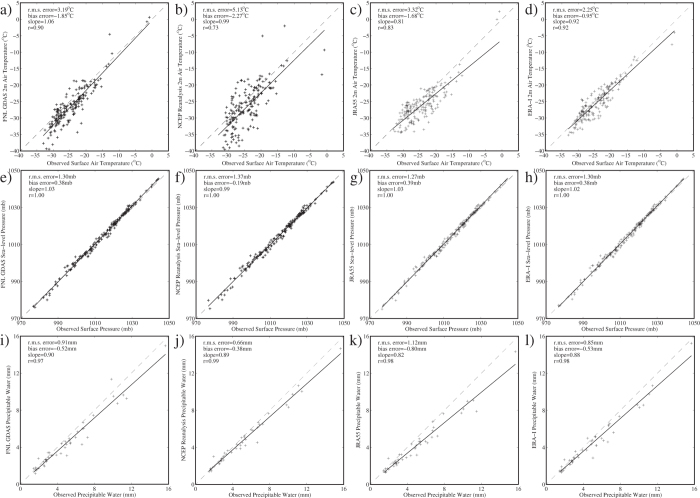
Comparison of observed and model meteorological parameters at the North Pole and Svalbard 1 December 2015–14 January 2016. Scatterplots of the observed surface air temperature at the North Pole, derived from the 7 meteorological buoys, with that from the: (**a**) final analysis of the NCEP Global Data Assimilation System, (**b**) the NCEP Reanalysis, (**c**) The JRA55 Reanalysis and (**d**) the ERA-I Reanalysis. Scatterplots of the observed surface pressure at the North Pole, derived from the 7 meteorological buoys, with that from the: (**e**) final analysis of the NCEP Global Data Assimilation System, (**f**) the NCEP Reanalysis, (**g**) the JRA55 Reanalysis and (**h**) the ERA-I Reanalysis. Scatterplots of the observed precipitable water, derived from radiosonde data at Ny Alesund, with that from the: (**i**) final analysis of the NCEP Global Data Assimilation System, (**j**) the NCEP Reanalysis, (**k**) the JRA55 Reanalysis and (**l**) the ERA-I Reanalysis. Error statistics for the fits are shown.

Time series of the surface air temperature and sea-level pressure at the North Pole as well as the precipitable water at Ny Alesund were extracted from the FNL-GDAS and the three reanalyses for the period 1 December 2015 to 14 January 2016. Scatterplots of the observed versus model data are shown in Fig. 3. In general the quality of the fit was better for the surface pressure as compared to the surface air temperature and precipitable water. With respect to the surface air temperature, the JRA55 and ERA-I Reanalyses and the FNL-GDAS had lower root mean square and bias errors and higher correlation coefficients as compared to the NCEP Reanalyses. In addition, all models were too cold with bias errors of ~1–2 °C. The NCEP and ERA-I Reanalyses had the lowest root mean square and bias errors for the precipitable water at Ny Alesund.

**Figure 4:**
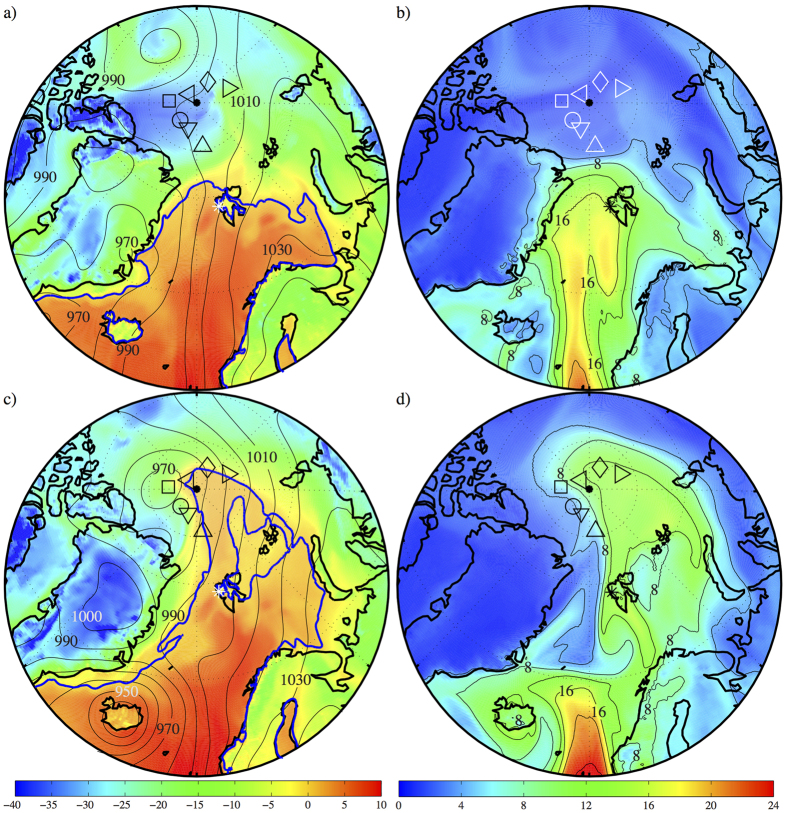
Meteorological conditions during the 2015 midwinter warming. The sea-level pressure (mb-contours) and surface air temperature (contour-^o^C) at 06 UTC on: (**a**) 29 December and (**c**) 30 December 2015. The precipitable water (mm-contours and shading) at 06 UTC on: (**b**) 29 December and (**d**) 30 December 2015. In a) and c) the 0oC isotherm is shown in blue. The locations of the 7 reporting meteorological buoys in the vicinity of the North Pole are indicated. All fields from the final analysis of the NCEP Global Data Assimilation System. Figure produced using MATLAB R2013b (http://www.mathworks.com).

Figure 4 shows the sea-level pressure, surface air temperature, and the precipitable water from the FNL-GDAS at 06 UTC on 29 December (one day before the warming event) and 30 December (at the time of the warming). The corresponding fields at 06 UTC on 30 December from the JRA55, NCEP and ERA-I Reanalyses are shown in Supplementary Figure 2. There is good overall agreement between the models, however the lower horizontal resolution of the NCEP Reanalysis resulted in a more muted representation of the fine scale structure of the flow.

On the 29^th^, there was a pronounced zonal gradient in the sea-level pressure field between a low along the northeast Greenland coast and a high over northern Scandinavia. In such a situation, one would expect and indeed observed moist southerly flow over the Nordic Seas, the Fram Strait and the Barents Sea (Fig. 4a & b). In this case, the flow resulted in the northward advection of warm moist air that was captured in the Ny Alesund radiosonde data (Fig. 1 and Supplementary Figure 1). At this time, the warm air mass was as far north as Svalbard with the 0 °C isotherm situated over the northern Fram Strait and Barents Sea.

Twenty-four hours later, the low that had been over northeast Greenland was now situated to the west of the North Pole and had a central pressure of 965 mb. This movement and its depth had resulted in a further northward advection of the warm and moist air into the region near the pole (Fig. 4c & d). The 0 °C isotherm indicates that above freezing surface air temperatures were present in the warm sector of the low including in the vicinity of the North Pole. In particular, the mean above freezing temperature north of 85°N at this time was 0.7 °C.

For this event, the 0 °C isotherm also provides a useful diagnostic for the presence of the warm and cold fronts associated with the low^29^. The cold front, in this instance the boundary between the cold air to the south of the low and the warm air to its southeast, can be seen to be located between the two buoys, with ids 132472 and 6400749, that experienced different temperatures during the warming event (Fig. 2). The buoys with ids 6400473, 6400476 and 6400751 were in the warm sector of the low and, as seen in Fig. 2, experienced above freezing temperatures. The warm front, in this instance the boundary between the warm air and the cold air to the east of the low, was in the vicinity of these buoys. There is also evidence of the so-called ‘bent back’ warm front, near the low’s center where the warm front bends back towards the cold front^30^; in this case it was situated in the vicinity of the buoys with ids 6400474 and 6400477.

At 6 UTC on 31 December, all the models indicated a return to more seasonal surface air temperatures near the pole with the 0 °C isotherm retreating southwards to the vicinity of Svalbard (not shown).

The widespread and spatially contiguous area of above freezing temperatures extending from the latitude of Svalbard northwards was associated with a widespread reduction in sea ice cover. Supplementary Figure 3 shows the difference in daily mean sea ice concentration retrieved using the ARTIST algorithm and the AMSR-2 data^31^ between 30 December and 29 December 29 2015, i.e. during the period of maximum warming at the pole. Along the Fram Strait’s marginal ice zone as well as over the Barents Sea, there was widespread reduction in sea ice concentration. In addition, there was a region deep within the ice pack in the vicinity of 82°N 35°E where there was a reduction in ice cover. To place this reduction in context, the difference in daily mean sea ice concentration between successive days over the period December 1-January 15 2002–2016, the years for which AMSR-E/AMSR-2 data is available, was calculated. The resulting histogram is also shown in Supplementary Figure 3 and indicates that the one-day sea ice loss during the 2015 midwinter warming event occurs less then 1% of the time.

The frontal boundaries of extra-tropical cyclones are preferred locations for precipitation^29^,^30^. Given the above freezing temperatures that occurred during this event, it is therefore possible that it was raining in the vicinity of pole during the event. Supplementary Figure 4 shows the forecast snowfall and rainfall rate at the time of the warming event from the FNL-GDAS. As expected, much of the precipitation associated with the low was in the form of snow. However in the vicinity of the warm front and the ‘bent back’ warm front, rainfall was forecast to have occurred.

On 30 December, all models indicated the presence of a deep low, central pressure < 950 mb, centered over Iceland (Fig. 4 & Supplementary Figure 2). This second low was also associated with another pulse of precipitable water that is clearly distinct from that associated with the warming at the pole (Fig. 4d). This low was responsible for flooding in North England and Scotland and was named ‘Storm Frank’ by the UK Met Office^32^. It originated over the Texas on 26 December where it was associated with a deadly outbreak of tornados^33^.

**Figure 5:**
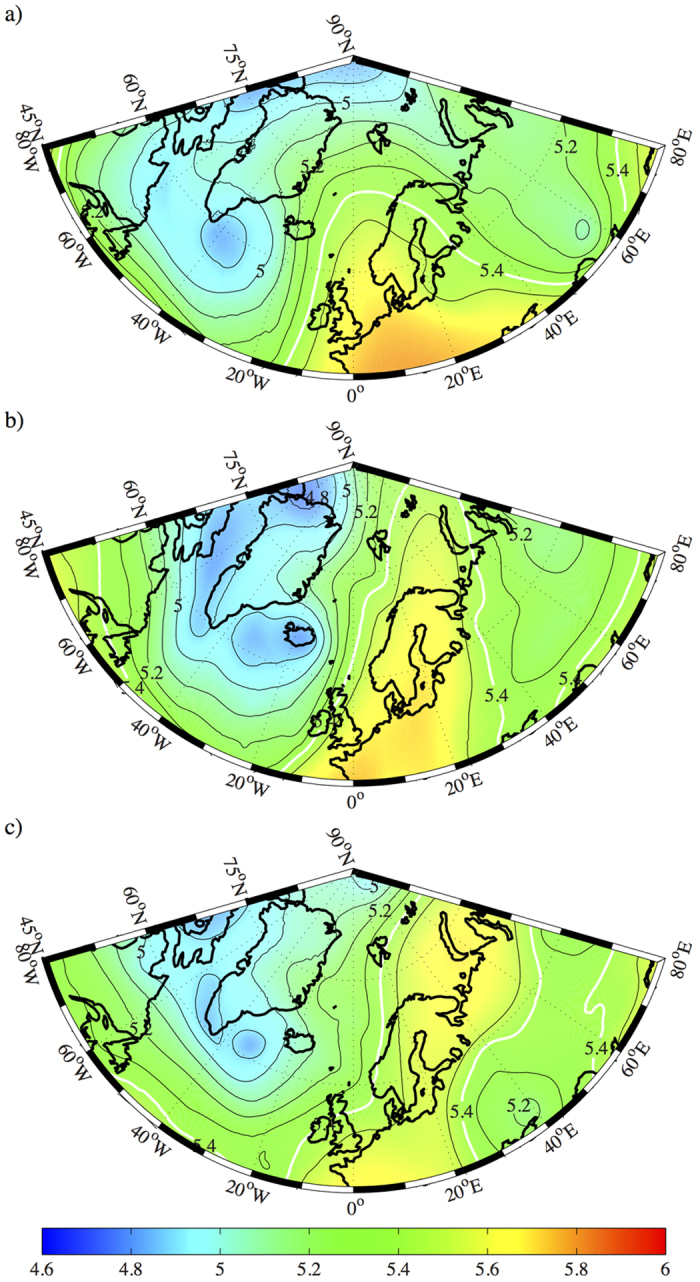
Evolution of the mid-tropospheric flow during the 2015 midwinter warming. The 500 mb geopotential height (km-contours and shading) at 06 UTC on: (**a**) 28 December 2015, (**b**) 30 December 2015 and (**c**) 1 January 2016. The 5.6 km isocontour, an indicator of the location of the jet stream and the polar vortex, is indicated by the thick white contour. All fields from the final analysis of the NCEP Global Data Assimilation System. Figure produced using MATLAB R2013b (http://www.mathworks.com).

There was, however, a connection between the two storms in that both were associated with a large-amplitude planetary wave^14^ that was located over the North Atlantic during late December 2015. This wave and its evolution can be seen in Fig. 5 where the 500 mb geopotential height (i.e. height of the 500 mb pressure surface) is shown at 06 UTC on 28 December and 30 December 2015 as well as 1 January 2016. The 5.6 km geopotential height contour provides a useful indicator for the high speed upper-level winds known as the jet stream as well as denoting the boundary of the polar vortex^7^. On the 28^th^, the trough of this wave, the region of low geopotential height, extended southeastwards from the Labrador Sea. The corresponding ridge, the region of high geopotential height, was situated over central Europe extending northwards towards the Nordic Seas as did the jet stream. By the 30^th^, the ridge along with the jet stream and the polar vortex was displaced to the northeast of Svalbard. As a result of this extension, there was a pronounced zonal gradient in geopotential height across the Nordic Seas that was associated with southerly flow that advected warm and moist air towards the pole (Fig. 4). By 1 January 2016, the ridge had weakened and moved off towards the east with the jet stream retreating southwards.

**Figure 6:**
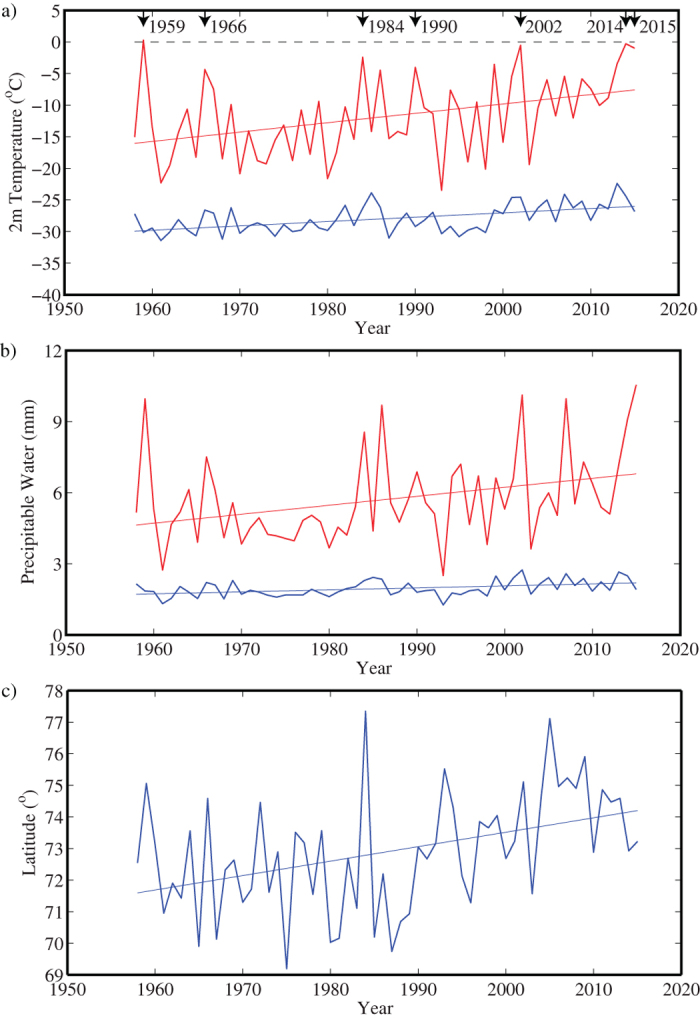
Mean and extreme meteorological conditions in the vicinity of the North Pole during December 1958–2015 from the JRA55 Reanalysis. Time series of mean (blue curves) and 99th percentile (red curves) for: (**a**) surface air temperature (^o^C), (**b**) precipitable water (mm) and (**c**) the latitude of the 0 ^o^C isotherm in the vicinity of Fram Strait. In a) and (**b**), years during which the 99th percentile 2 m air temperature approached 0 °C are indicated. Linear least squares fits to the time series are also shown. All trends are statistically significant at the 95th percentile confidence interval.

To investigate the uniqueness of the 2015 event, the JRA55 Reanalysis was used to identify the mean and 99^th^ percentile surface air temperature and precipitable water in the region north of 85^o^N for each December from 1958–2015. The results of this calculation are shown in Fig. 6 and clearly reveal an increasing trend in the mean and 99^th^ percentile values for both the surface air temperature and the precipitable water since the late 1950 s. All trends are statistically significant at the 95^th^ percentile confidence interval using a test that takes into account the reduced degrees of freedom arising from temporal autocorrelation of geophysical time series^34^. For both fields, the positive trend in the extreme values was greater then that for the mean values. For the surface air temperature this ratio was ~2, while for the precipitable water it was ~4. The higher ratio for precipitable water is consistent with the idea that in a warming climate there is an intensification of the high latitude hydrological cycle resulting from the non-linear relationship between temperature and saturation specific humidity^35^. Supplementary Figures 4 and 5 indicate that similar behaviour was also observed in the NCEP and ERA-I Reanalyses.

This suggests that, in addition to a long-term warming of the North Pole region, there have been changes that result in a greater efficiency for cyclones to access the reservoir of warm and moist air that exists over the nearby Nordic Seas (Fig. 1) and transport it polewards leading to warmer and moister extreme events. Amongst the changes that may have contributed to this efficiency are reductions in the winter sea ice over the Greenland and Barents Seas that have moved this reservoir of warm and moist air closer to the pole. Figure 6 also shows the latitude of the 0 °C isotherm of the surface air temperature during December over Fram Strait from the JRA55 Reanalysis. The latitude of this isotherm is moving northwards at a statistically significant rate of ~0.5^o^/decade, a result that was also seen in the NCEP Reanalysis (Supplementary Figure 4).

A number of events during which the 99^th^ percentile surface air temperature was close to 0 °C can be seen in Fig. 6. It should be noted that the surface air temperature from the JRA55 Reanalysis has a cold bias of ~2 °C and so, the mean and extreme values are most likely underestimated. Most of these warming events were also associated with maxima in precipitable water. Supplementary Figures 4 & 5 confirm that these warming events were also captured in the NCEP and ERA-I Reanalyses.

The earliest of these events occurred in December 1959. The atmospheric circulation during this event, as represented in the JRA55, is shown in Supplementary Figure 6. It shares many characteristics with the 2015 event, including the presence of a deep extra-tropical cyclone to the west of the pole, southerly flow in the vicinity of the pole that advected warm and moist air into the region and a perturbed polar vortex with the presence of the jet stream in the vicinity of the pole. The JRA55 Reanalysis indicates that a warming event also occurred during December 2014. This event was also captured by the ERA-I Reanalysis but was not as well pronounced in the NCEP Reanalysis. An examination of the environmental conditions during this event indicated a high degree of commonality with the 1959 and 2015 events. The NCEP Reanalysis failed to capture it because of its lower horizontal resolution.

## Discussion

The characteristics of the late December 2015 warming event have been investigated with *in-situ* buoy and radiosonde data as well as operational analyses and reanalyses. Based on buoy data, the surface air temperature at the North Pole rose by ~26 °C during a 24 hour period ending at 06 UTC on 30 December. The warming was associated with a drop in surface pressure of ~24 mb. Indeed, the central sea-level pressure of the low at 06 UTC on 30 December was the lowest sea-level pressure attained in the JRA55 in the vicinity of the pole during December 1958–2015 (not shown). There was considerable spatial variability in the model surface air temperature in the vicinity of the pole during this event that was associated with the organization of the extra-tropical cyclone that was responsible for the warming. However the model fields all are consistent with a short duration event.

Based on the available buoy data, the surface air temperature at the pole did not rise above freezing, although above freezing temperatures were observed at the buoys that were situated in the warm sector of the cyclone close to the pole. The uncertainty in the surface air temperature data from the IABP buoys is on the order of 0.5 °C and it is possible that the temperatures were close to freezing rather then being above 0 °C. However the 2 m air temperature field from the FNL-GDAS indicated that there was a large spatially coherent region of above freezing temperatures in the vicinity of the North Pole on the 30^th^ that is in broad agreement with the buoy data. The boundaries of this region that are associated with the cyclone’s warm and cold fronts are consistent with the observed variability in the IABP data.

The surface warming associated with the event was coincident with a widespread reduction in sea ice cover in the region. Care must be taken in interpreting the one-day sea ice loss to the warming during the event. There are issues with the microwave retrieval of sea ice at low concentrations as well in the presence of water on ice, that may have occurred during this event due to the presence of rain, that could have influenced the one-day ice loss observed during this event^36^. In addition, the mechanical movement of sea ice by the surface wind field could also have contributed to the loss. However, the lack of large-scale regions of enhanced ice cover, as would be required to balance the mechanically-forced divergence, suggests that this process was not widespread. In any event, the reduction in sea ice concentration between the 29^th^ and 30 in the region of interest was the largest during the midwinter period in the 15 year long record in the AMSR-E/AMSR-2 dataset.

The cyclone responsible for the warming also advected moisture polewards. On Svalbard, the magnitude of the moisture pulse exceeded the 99^th^ percentile value during midwinter. The forecast fields from the FNL-GDAS predicted that this event resulted in rainfall in the vicinity of the North Pole. Care must be taken as the precipitation field from an analysis or reanalysis is not strongly constrained by observations and is therefore highly dependent on the characteristics of the underlying model^27^.

In addition to the advective surface warming that occurred during the event, the elevated levels of water vapour throughout the lower troposphere that were also present most likely warmed the surface by increasing the downwelling longwave radiation^15^,^17^. Models continue to have issues with this process at high latitudes as as a result of an inability to correctly represent the cloud fraction and cloud phase^37^,^38^. This issue may contribute to the cold bias observed in the model surface air temperature at the North Pole.

Contrary to media reports^1^,^2^,^3^, the cyclone responsible for the warming event at the pole was not the one responsible for the severe weather in Texas and the United Kingdom that preceded this event. There was however a connection in that all this severe weather was associated with a highly perturbed polar vortex that brought the jet stream close to the pole.

It was also shown that these warming events at the pole occur once or twice each decade with an event in 2014 and the earliest event that has been so far identified taking place in 1959. The 1959, 2014 and 2015 events share a number of common characteristics including the presence of a deep extra-tropical cyclone in the vicinity of the pole that transported warm and moist air towards the pole. All events also were associated with a highly perturbed polar vortex that brought the jet stream close to the pole.

The December 99^th^ percentile surface air temperature and precipitable water in the vicinity of the North Pole have been increasing at a rate higher than that for mean values. This ratio is higher for precipitable water, a result that is consistent with an intensification of the high latitude hydrological cycle in a warming climate^35^. It is proposed that this enhanced trend in extreme warmth and moisture at the pole is associated with the loss of winter sea ice from the adjoining Greenland and Barents Seas that is moving the reservoir of warm and moist air that exists over the Nordic Seas closer to the pole allowing for a greater efficiency of cyclones in transporting this heat and moisture polewards.

Such warming events and the associated occurrence of rainfall at high northern latitudes can lead to significant stresses on Arctic infrastrcuture^18^ as well mammalian herbivoiries^19^,^20^,^39^ that inhabit the Arctic Islands, These stresses may become more pronounced as the occurrence of these midwinter warming events become more frequent as the region transitions towards the occurrence of warmer and wetter winters.

## Methods

During December 2015, there were 7 meteorological buoys within 500 km of the North Pole (Fig. 1 & Table 1). These buoys provide hourly surface air temperature and pressure data that are available in near real time through the International Arctic Buoy Porgram (IABP)^40^. The root mean square temperature error for the buoys is on the order of 0.5 °C^24^. For the period 1 December 2015–14 January 2016, this data was interpolated to generate an estimate of the surface air temperature and pressure at the North Pole (Fig. 1). A linear interpolation scheme was used and the issue of converging meridians at the pole^41^ was taken into account through the use of a rotated co-ordinate system that placed the North Pole at 45^o^N, 45^o^W.

Data from the radiosonde site at Ny Alesund on Svalbard (78.91^o^N, 11.93^o^E) was also used to characterize the vertical structure of the moisture plume associated with the warming event. Please refer to Fig. 1 for the location of the radiosonde site. During December 2015, the data was typically available once-daily at 12 UTC. Data over the entire period of availablilty, December 1993-January 2016, was used to calculate mean as well as 95^th^ and 99^th^ percentile values.

In meteorology, the term analysis refers to the outcome of the process of data assimilation that generates a representation of the state of the atmosphere at a given time that is suitable for use as initial conditions in subsequent forecasts^42^. Operational analyses suffer from changes over time that render them unsuitable for longer term studies^43^. Retrospective analyses or reanalyses correct this fault by using a modern data assimilation system to assimilate all available historical observations to generate homogeneous time series of the state of the atmopshere that allow one to place current events in a longer-term context^43^. The precipitation field from an analysis or renalysis is not strongly constrained by observations and is therefore influenced by the characteristics of the model used in the data assimilation process^27^. In addition, it is typically stored as an average over the period of the short-term forecast used to advance the analysis or reanalysis^27^. The surface air temperature from a numerical model is typically expressed as the temperature at a height of 2 m above the surface.

The final operational analysis from the National Centers for Environmental Prediction (NCEP) Global Data Assimilation System^25^ (FNL-GDAS) is available in near real time at a horizontal resolutuion of 0.25^o^ on 26 pressure levels with 25 mb spacing below 900 mb. Three reanalysis products, which are all available on a near real time basis, were also used. The JRA55 Reanalysis^26^ from the Japanese Meteorological Agency has a horizontal resolution of 1.25^o^ and extends back to 1958. The NCEP Reanalysis^27^ has a horizontal resolution of 1.875° and extends back to 1948. However issues with sparseness of data at high northern latitudes exist up to the establishment of the global radiosonde network in 1958^22^. For this reason, we restricted our investigation of warming events at the North Pole to the time period 1958–2015. The ERA-I Reanalysis^28^ from the European Center for Medium-Range Weather Forecasts has a horizontal resolution of 0.75^o^ and extends back to 1979. All datasets are available at a temporal resolution of 6 hours.

The daily mean sea ice concentration derived from the AMSR-E/AMSR-2 instruments using the ARTIST algorithm^31^ was used to characterize the reduction in ice cover associated with the 2015 midwinter warming event. This instrument has additonal high frequency microwave channels that allow for higher spatial resolution retreivals as compared to the SSM/I^31^. This data is available at a horizontal resolution of 6.25 km for the period from 2002–2016. Comparsions of the results of the ARTIST algorithm with the NASA-Team 2 and Bootstrap algorithms show good agreement^31^.

## Additional Information

**How to cite this article**: Moore, G. W. K. The December 2015 North Pole Warming Event and the Increasing Occurrence of Such Events. *Sci. Rep.* **6**, 39084; doi: 10.1038/srep39084 (2016).

**Publisher's note:** Springer Nature remains neutral with regard to jurisdictional claims in published maps and institutional affiliations.

## Supplementary Material

Supplementary Information

## Figures and Tables

**Table 1:** Details of the buoys in the vicinity of the North Pole that were reporting during the December 2015 warming.

ID	Type	Latitude on Dec 30 2015	Longitude on Dec 30 2015	Distance from North Pole on Dec 30 2015
132472	Airborne Expendable Ice Buoy	87.5^o^N	42.4^o^W	274.1 km
6400476	Ice Beacon	87.5^o^N	153.6^o^E	281.5 km
6400477	Ice Beacon	87.0^o^N	94.9^o^W	329.3 km
6400474	Ice Beacon	88.7^o^N	137.9^o^W	145.6 km
6400751	Ice Beacon	85.6^o^N	8.7^o^E	494.2 km
6400749	Ice Beacon	87.3^o^N	19.2^o^W	302.1 km
6400473	Ice Beacon	86.4^o^N	116.3^o^W	403.3 km

Data available at iabp.apl.washington.edu.
