# Supplementary material for: The December 2015 North Pole Warming Event and the Increasing Occurrence of Such Events
Source: Sci Rep. 2016 Dec 15;6:39084. doi: 10.1038/srep39084 (PMC5157030; doi:10.1038/srep39084)
Supplement: Supplementary Information [file srep39084-s1.pdf]

The December 2015 North Pole Warming Event and the Increasing Occurrence of Such Events

G.W.K. Moore
Department of Physics
University of Toronto
Toronto, Ontario Canada

Supplementary Material

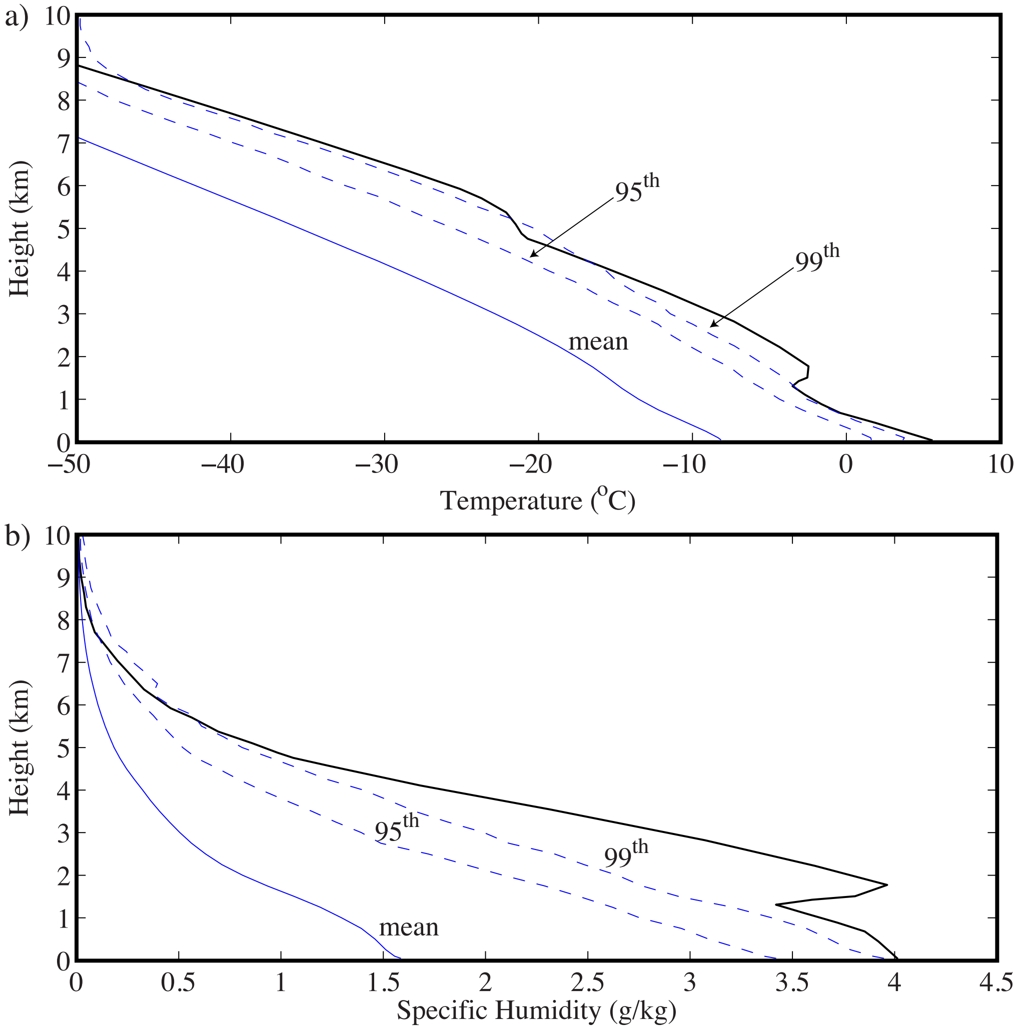

Supplementary Figure 1) Vertical profiles of: a) temperature (ºC) and specific humidity (g/kg) at 12 UTC on 29 December 2015 at Ny Alesund. Mean, 95th and 99th percentile profiles are also shown that are based on available data for the period 1 December -14 January during the years 1994-2016.

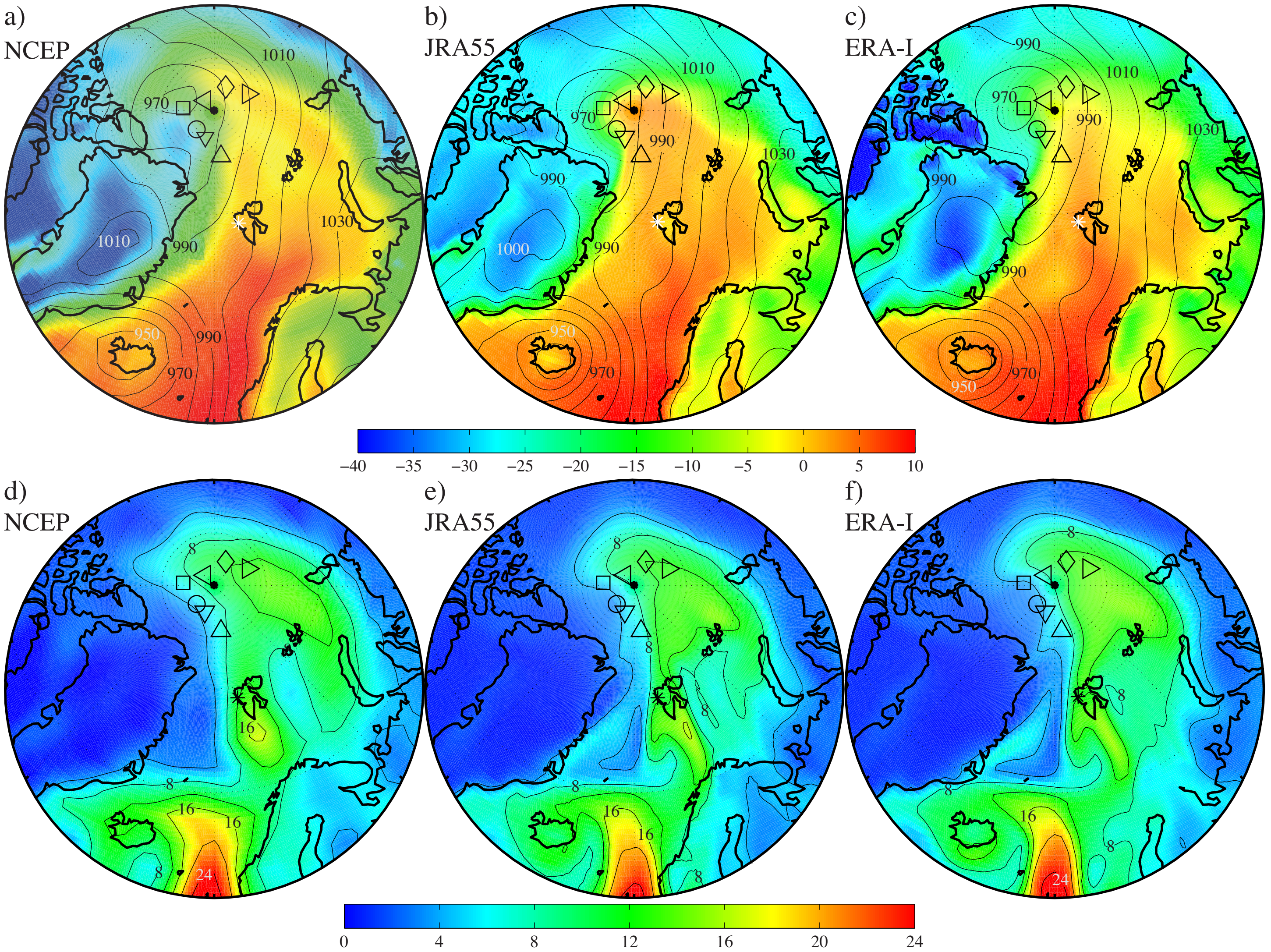

Supplementary Figure 2) Meteorological conditions during the late December 2015 warming. The sea-level pressure (mb-contours) and surface air temperature (shading-ºC) at 06 UTC on 30 December 2015 from: a) the NCEP Reanalysis, b) the JRA55 Reanalysis and c) the ERA-I Reanalysis. The precipitible water (mm-contours and shading) at 06 UTC on 30 December 2015 from: d) the NCEP Reanalysis, e) the JRA55 Reanalysis and f) the ERA-I Reanalysis. The locations of the 7 reporting meteorological buoys in the vicinity of the North Pole are indicated as is the radiosonde station at Ny Alesund. Figure produced using MATLAB R2013b (http://www.mathworks.com).

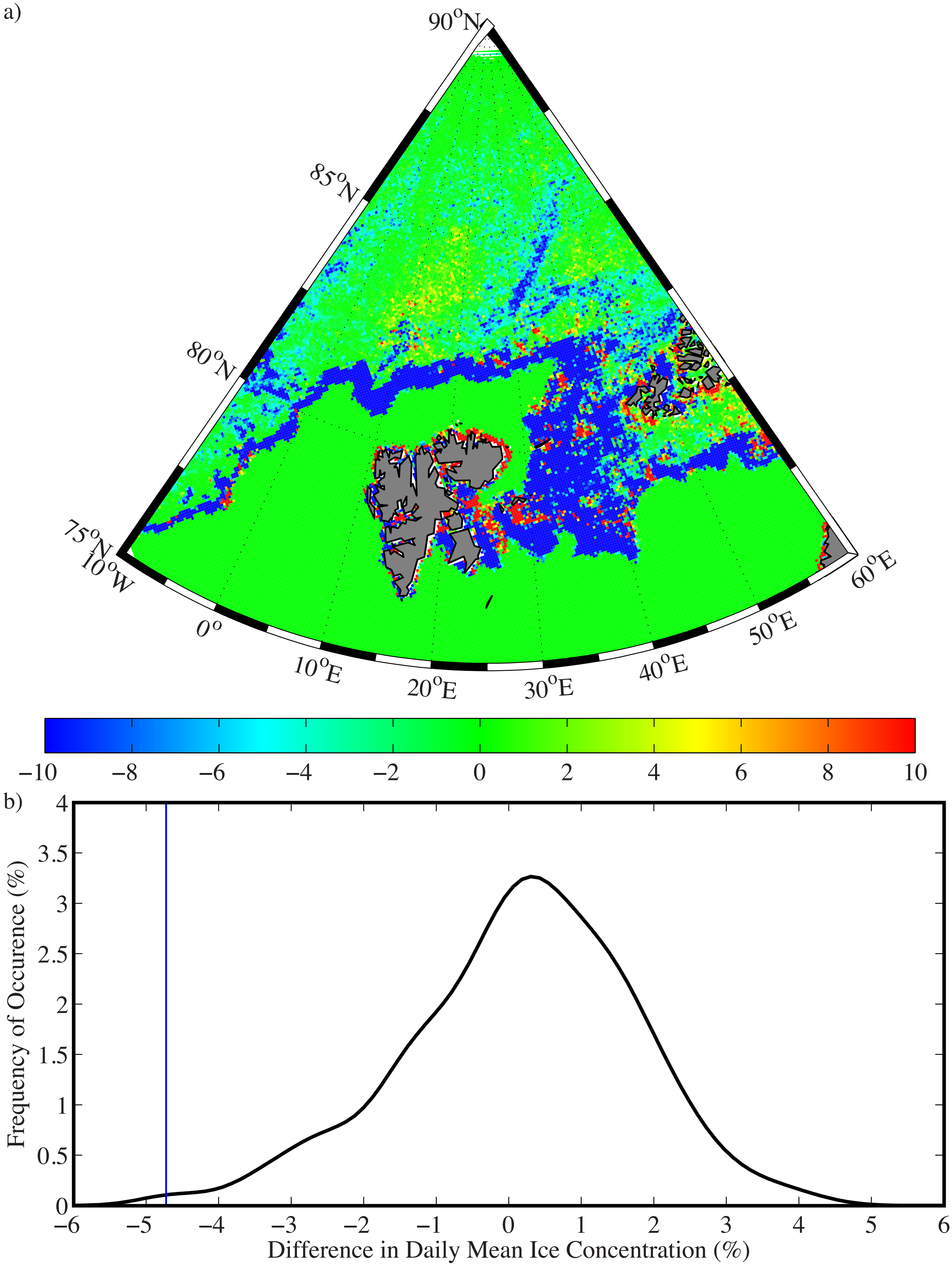

Supplementary Figure 3) The difference in daily mean sea ice concentration (%) based on the AMSRE/AMSRE2 data. a) The difference between December 29 and December 30 2015 in the region near Svalbard. b) The historgram of the differences over the region shown in a) for the period December 1 - January 15 for the years 2002-2016. The difference between December 29 and December 30 2015 is indicated by the vertical blue line. Figure produced using MATLAB R2013b (http://www.mathworks.com).

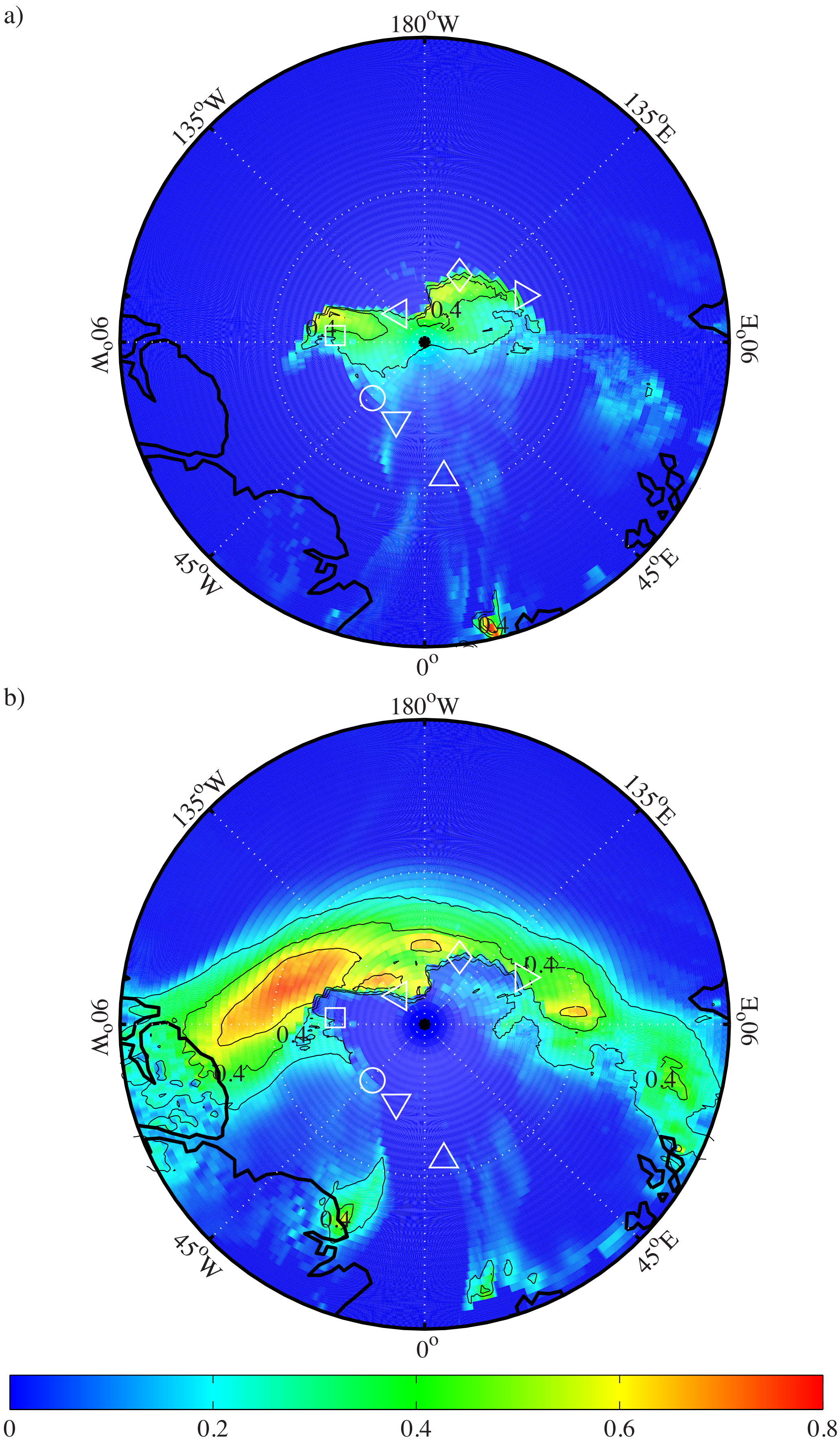

Supplementary Figure 4) Precipitation in the vicinity of the North Pole during the late December 2015 warming. a) The rainfall rate (mm/hr) and b) the snowfall rate (mm/hr water equivalent) at 06 UTC on 30 December 2015. The locations of the 7 reporting meteorological buoys in the vicinity of the North Pole are indicated. All fields are from the final analysis of the NCEP Global Data Assimilation System. Figure produced using MATLAB R2013b (http://www.mathworks.com).

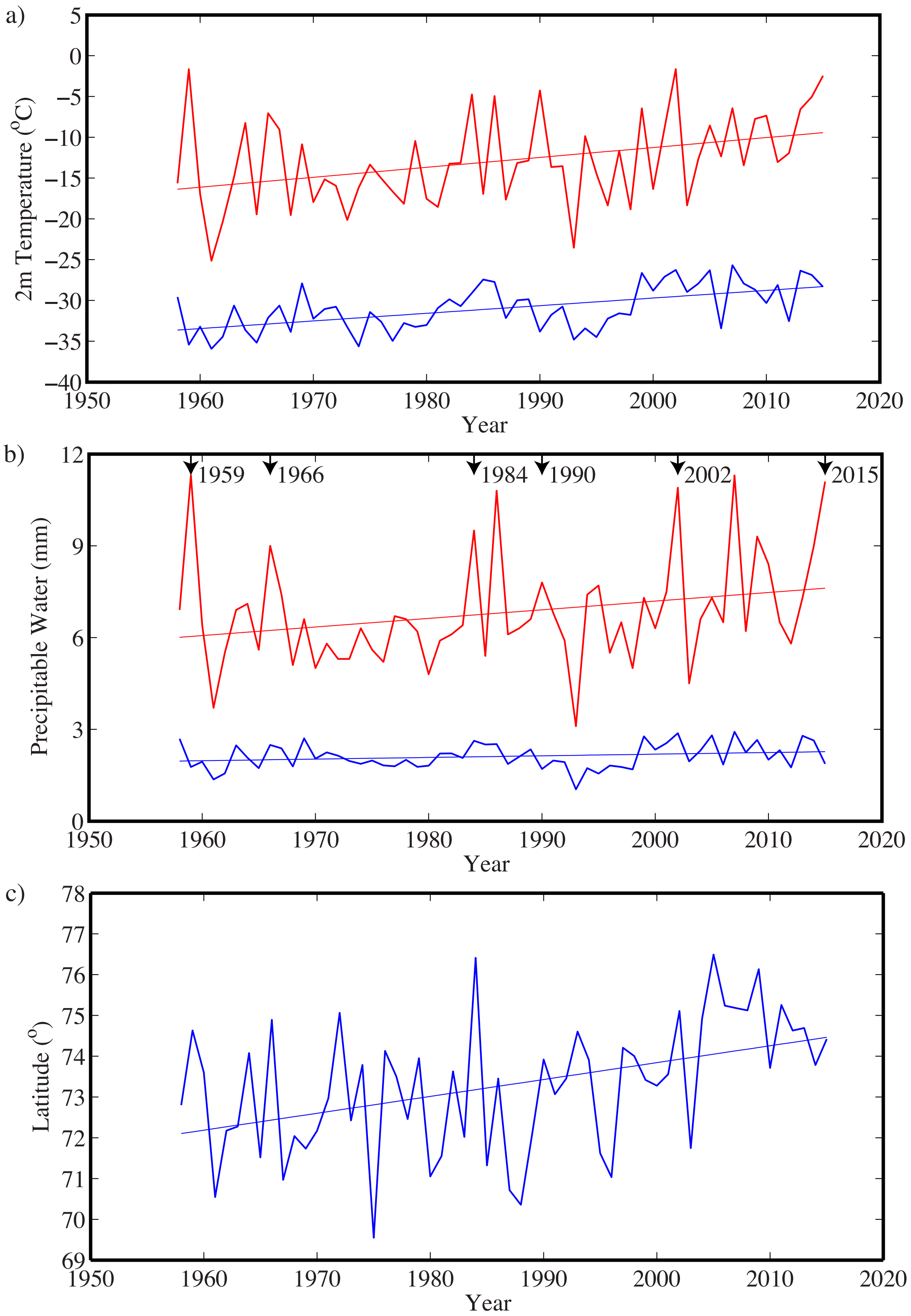

Supplementary Figure 5) Mean and extreme meteorological conditions in the vicinity of the North Pole during December 1958-2015 from the NCEP Reanalysis. Time series of mean (blue curves) and 99th percentile (red curves) for: a) surface air temperature (^{o}C), b) precipitable water (mm) and c) the latitude of the 0°C isotherm in the vicinity of Fram Strait. Linear least squares fits to the time series are also shown. All trends are statistically significant at the 95th percentile confidence interval.

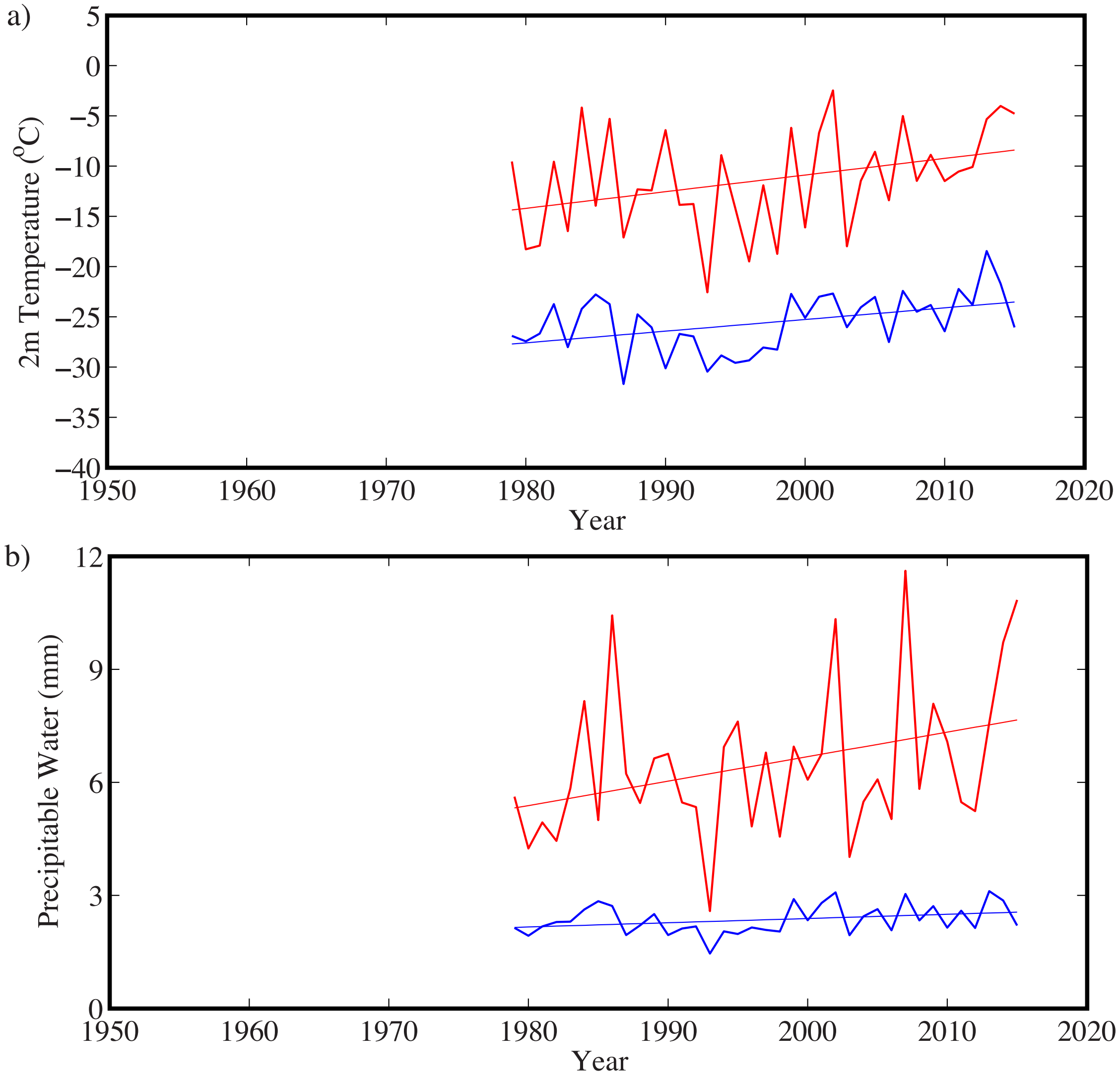

Supplementary Figure 6) Mean and extreme meteorological conditions in the vicinity of the North Pole during December 1979-2015 from the ERA-I Reanalysis. Time series of mean (blue curves) and 99th percentile (red curves) for: a) surface air temperature (°C), b) precipitable water (mm) and c) the latitude of the 0°C isotherm in the vicinity of Fram Strait.

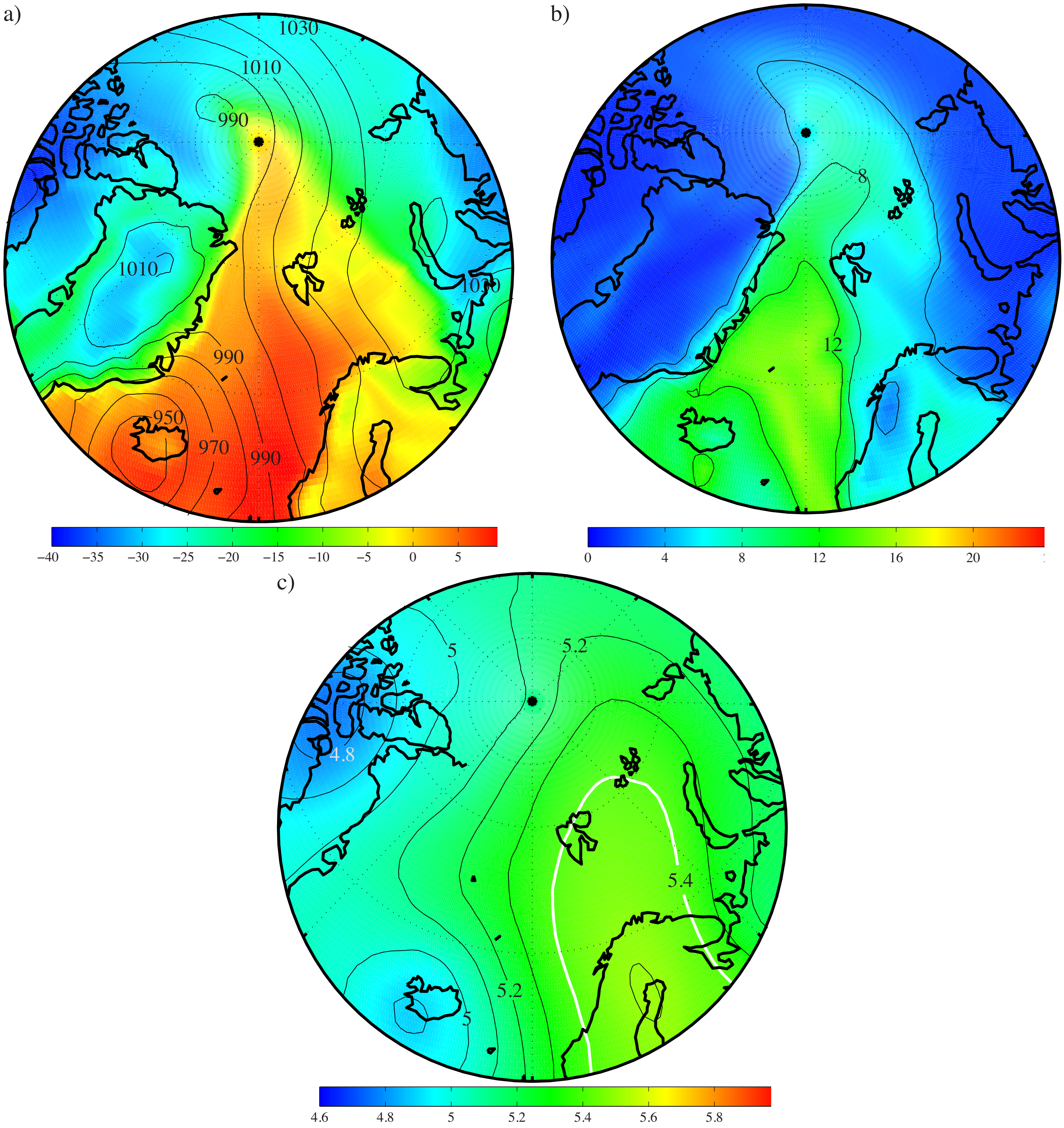

Supplementary Figure 7) Meteorological conditions during the December 1959 warming. The: a) sea-level pressure (mb-contours) and surface air temperature (contour-°C), b) the precipitble water (mm-contours and shading) and c) the 500mb geopotential height (km-contours and shading) at 00 UTC on 3 December 1959. In c) the 5.6 km isocontour, an indicator of the location of the jet stream, is indicated by the thick white contour. All fields from the JRA55 Reanalysis. Figure produced using MATLAB R2013b (http://www.mathworks.com).
